# MicroED structure of the C11 cysteine protease clostripain

**DOI:** 10.1016/j.yjsbx.2024.100107

**Published:** 2024-07-06

**Authors:** Yasmeen N. Ruma, Guanhong Bu, Johan Hattne, Tamir Gonen

**Affiliations:** a Howard Hughes Medical Institute, University of California, Los Angeles, Los Angeles, CA 90095, United States; b Department of Biological Chemistry, University of California, Los Angeles, Los Angeles, CA 90095, United States; c Department of Physiology, University of California, Los Angeles, Los Angeles, CA 90095, United States

**Keywords:** Clostripain, Cysteine protease, Microcrystal electron diffraction, MicroED, Cryo-EM

## Abstract

Clostripain secreted from *Clostridium histolyticum* is the founding member of the C11 family of Clan CD cysteine peptidases, which is an important group of peptidases secreted by numerous bacteria. Clostripain is an arginine-specific endopeptidase. Because of its efficacy as a cysteine peptidase, it is widely used in laboratory settings. Despite its importance the structure of clostripain remains unsolved. Here we describe the first structure of an active form of *C. histolyticum* clostripain determined at 2.5 Å resolution using microcrystal electron diffraction (MicroED). The structure was determined from a single nanocrystal after focused ion beam milling. The structure of clostripain shows a typical Clan CD α/β/α sandwich architecture and the Cys231/His176 catalytic dyad in the active site. It has a large electronegative substrate binding pocket showing its ability to accommodate large and diverse substrates. A loop in the heavy chain formed between residues 452 and 457 is potentially important for substrate binding. In conclusion, this result demonstrates the importance of MicroED to determine the unknown structure of macromolecules such as clostripain, which can be further used as a platform to study substrate binding and design of potential inhibitors against this class of peptidases.

## Highlights

• The structure of active clostripain was determined using microcrystal electron diffraction (MicroED).
• The structure was obtained from two nanocrystals after focused ion beam milling.
• The structure identifies a loop for substrate binding.

## Graphical abstract

**Figure:**
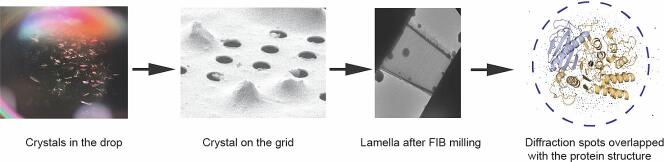

## Introduction

*Clostridium histolyticum*/*Hathewaya histolytica* is a gram-positive pathogenic bacterium that is known to cause local necrosis in human muscles, organs and connective tissues. *C. histolyticum* can secrete five different kinds of potent exotoxins that includes proteinases and collagenases (Nishida and Imaizumi, 1966, Oakley and Warrack, 1950). These toxins can cause proteolysis and degradation of cells (Hatheway, 1990), thus leading to systemic toxemia (Durmaz et al., 2000) and eventually death if left untreated (Flores-Diaz and Alape-Giron, 2003). In addition to the collagenases and peptidases, a cysteine-activated protease, known as clostripain was also isolated from the culture filtrates of *C. histolyticum* (Labrou, 2013, Kocholaty et al., 1938)*.* However, there is no report on the proteolytic effect of clostripain on human cells yet. The protease is mainly used *in vitro* as an important research tool for protein sequencing and peptide fragment condensation, and has applications in human islet isolation (Ståhle et al., 2015).

Clostripain is the archetypal member of the C11 family of the Clan CD of cysteine endopeptidases (Rawlings et al., 2012). Since its discovery in 1938, there has been ongoing research on clostripain (Kembhavi et al., 1991, Labrou and Rigden, 2004, Ullmann and Jakubke, 1994, Witte et al., 1996) and the homologue proteases of the C11 family (Manabe et al., 2010, McLuskey et al., 2016, McLuskey and Mottram, 2015). Although the proteolysis effect of clostripain on the pathogenic process is not yet known, there have been reports of the involvement of other C11 proteases in the pathogenicity of disease in humans. For example, a clostripain-like protease secreted from the commensal pathogen *Clostridium perfringens* was reported to promote macrophage phagocytosis by degradation of host neutrophils (Guzik et al., 2007). Another peptidase from the same family, fragipain from *Bacteroides fragilis* was reported to cause sepsis in mice (Choi et al., 2016), thus making this family of proteases an interesting group to explore.

The members of the Clan CD cysteine peptidases are classified mainly based on similar structural features and function, rather than sequence homology (Labrou and Rigden, 2004, Rawlings et al., 2012). These proteases have a highly conserved histidine/cysteine catalytic dyad and use an active cysteine to cleave protein peptide bonds (McLuskey and Mottram, 2015). The first structure determined from this family was of PmC11, from *Parabacteroides merdae* (McLuskey et al., 2016), which was used as the model to study the structure–function relationship of C11 peptidases. From then on, crystal structures of clostripain-like proteases from different bacteria have been determined either in unbound form (Gonzalez-Paez et al., 2019, McLuskey et al., 2016) or in complex with their inhibitors (Roncase et al., 2019, Roncase et al., 2017) by X-ray diffraction. However, clostripain which has a high specificity for arginine and requires calcium ions for activation (Kembhavi et al., 1991, Witte et al., 1994) remained without an experimental structure probably due to its low yield in heterologous host expression systems (Manabe et al., 2010) prohibiting large scale crystal screening.

In this study, we have determined the first experimental structure of *Clostridium histolyticum* clostripain in its active state at 2.5 Å resolution by the cryogenic electron microscopy (Cryo-EM) method microcrystal electron diffraction (MicroED). MicroED is a robust method used to determine structures of different samples including small molecules, peptides, natural products and proteins using vanishingly small crystals (Mu et al., 2021, Nannenga and Gonen, 2019). The structure consists of a typical Clan CD α/β/α sandwich architecture and the Cys231/His176 catalytic dyad in the active site. A large electronegative cavity was identified as the substrate binding pocket. The structure allowed us to identify a loop between residues 452 and 457 that may be important for substrate binding. This study demonstrates MicroED’s ability to deliver structures that were not attainable by other methods even when the sample is prohibitively limiting for large-scale crystal screening.

## Results and discussion

### Structure determination

Initial clostripain crystals were formed as needles within 2 to 3 days at 20 °C in presence of 0.2 M ammonium phosphate monobasic, 0.1 M TRIS pH 8.5, 50 % v/v 2-methyl-2,4-pentanediol. These crystals were difficult to reproduce and so seeding was attempted. Seeding stock made from these crystals was used to initiate crystal growth in several different conditions. Small plate-shaped crystals were observed in 0.2 M ammonium acetate, 0.1 M Na-citrate tribasic dihydrate pH 5.6, 30 % w/v PEG 4000 within a day of seeding.

**Fig. 1:**
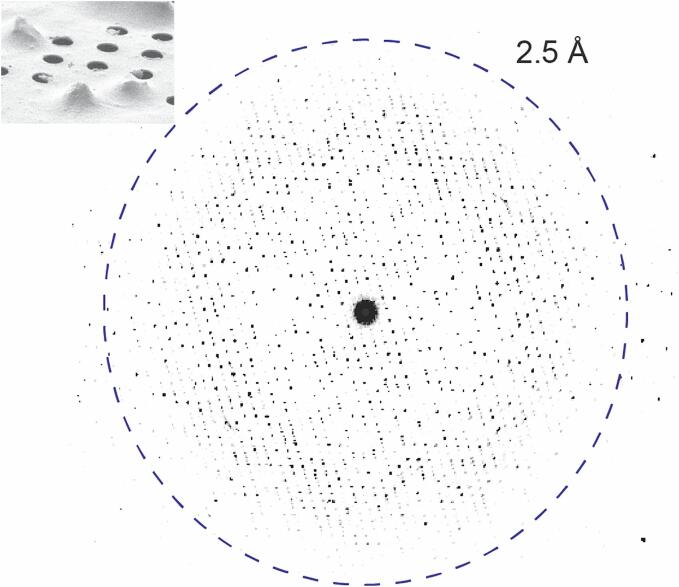
Representative MicroED pattern of a clostripain lamella collected by continuous rotation. Inset shows a FIB/SEM image of clostripain crystals on a Quantifoil holey carbon grid. The side edge length of the crystals was approximately 1 µm.

The crystals were approximately 1 µm in size, thus making them unsuitable for X-ray diffraction. They were also not directly suitable for MicroED analysis as they were too thick to be traversed by electrons in the transmission electron microscope (TEM). The crystals were transferred to EM grids, plunge-frozen in liquid ethane and stored in liquid nitrogen and thinned using a cryogenic focused ion beam (FIB) in a scanning electron microscope (SEM) to a thickness suitable for MicroED (Martynowycz et al., 2019). With this approach the crystal lamellae ∼300 nm in thickness were produced (Fig. 1). The grids containing the milled lamellae were then transferred to the TEM operating under cryogenic conditions. The Thermo Fisher EPU and Velox packages were used for locating the lamellae and screening for diffraction quality. The MicroED data were collected in counting mode with a Thermo Fisher Falcon 4i direct electron detector using the continuous rotation method (Nannenga and Gonen, 2014) and an energy filter with slit size of 20 eV.

**Fig. 2:**
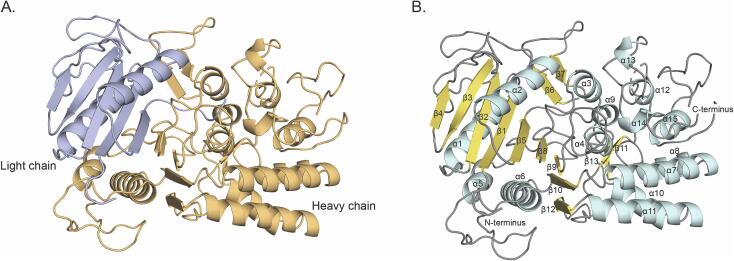
MicroED structure of clostripain. (A) Quaternary structure of clostripain. The light chain is shown in purple and heavy chain is shown in orange. (B) Tertiary structure of clostripain. α-Helices are shown in cyan, β-strands in yellow and the loops in gray. The N- and C-termini, α-helices and β-strands are all labelled. The helices and strands are numbered based on the sequence starting from the N-terminus.

MicroED datasets were collected using a total of nine lamellae and the dataset from the best diffracting lamellae were processed (Fig. 1) to a resolution of 2.5 Å and an overall completeness of 87 % (Table 1). The data were processed and refined as detailed in the methods section. Clostripain was crystallized in the space group *P* 2 2_1_ 2_1_ with cell dimensions of a = 65.79 Å, b = 106.07 Å, c = 149.28 Å and α = β = γ = 90°. The AlphaFold model (Model ID: AF-A0A4U9RR22-F1) (Jumper et al., 2021) for *C. histolyticum* clostripain proenzyme was used to phase the data using molecular replacement. In the sequence of clostripain proenzyme, residues 1–27 represent the signal peptides, 28–50 represent the pro-peptide, 51–181 is the light chain, 182–190 is the linker joining the light and heavy chain and 191–526 is the heavy chain of clostripain (Uniprot ID: P09870) (Fig. 2). The residues for the signal peptide and the pro-peptide were removed for phasing, as molecular replacement was not successful when they were left intact. During refinement, no density was observed for either the linker or residues 452–457 in the heavy chain suggesting that these residues form a flexible dynamic loop; for subsequent refinement this section of the protein was also removed. Following several rounds of refinement and manual building and rebuilding, the structure was finalized with R_work_/R_free_ = 24 %/27 % at 2.5 Å resolution, and with 96 % of the residues in the favorable region of the Ramachandran plot (Table 1). The final model in the asymmetric unit has a dimer of clostripain heterodimers comprising a light chain (residues 52–181) and a heavy chain (residues 191–526).

**Table 1:** MicroED data collection and refinement statistics.

**Data collection**	
Wavelength (Å)	0.0197
Resolution range (Å)	49.4–2.50 (2.56–2.50)
Space group	*P* 2 2 2
Unit cell (a, b, c) (Å)	65.79, 106.07, 149.28
(α, β, γ) (°)	90, 90, 90
Total reflections (#)	131,476 (9,401)
Unique reflections (#)	32,113 (2,318)
Multiplicity	4.1 (4.1)
Completeness (%)	86.8 (86.5)
Mean I/σ(I)	3.8 (1.4)
CC_½_	0.975 (0.615)
R_merge_	0.279 (0.815)
Wilson B-factor (Å^2^)	30.8

**Refinement**	
Resolution (Å)	49.4–2.50 (2.58–2.50).
Space group	*P* 2 2_1_ 2_1_
R_work_	0.238 (0.351)
R_free_	0.273 (0.376)
Average isotropic B-factor (Å^2^)	24.7
RMS-bonds (Å)	0.002
RMS-angles (°)	0.546
Ramachandran favored, allowed, outliers (%)	95.7, 3.88, 0.44
Clashscore	4.08

Values in parentheses indicate the highest resolution shell.

### Overall structure of clostripain

**Fig. 3:**
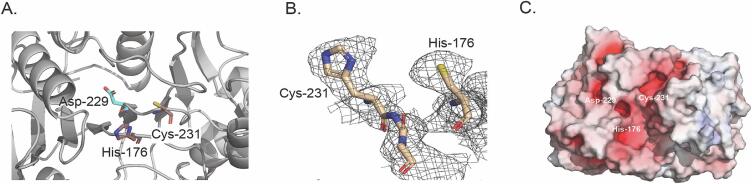
(A) Structure of clostripain highlighting the catalytic dyad, His176 and Cys231. The P1 specific substrate site Asp229 is presented in cyan. Nitrogen, oxygen and sulphur atoms are colored blue, red and yellow, respectively. (B) 2F_o_-F_c_ map (gray mesh) contoured at 1σ showing density for the active site residues. (C) Electrostatic surface potential of clostripain in the same orientation as A showing the same residues as A, where blue and red denote positively and negatively charged surface potential, respectively, contoured at ±10 kT/e.

Clostripain in its proenzyme form is a 59 kDa sized protein. Its polypeptide chain is composed of a heavy and a light chain that are connected by a linker region and held together by strong non-covalent forces. When a calcium ion activates the proenzyme, it undergoes auto-maturation by autocatalyzing the removal of the linker peptide at its two cleavage sites—Arg 181, part of the light chain (labelled in Fig. 3A and 3C), and Arg 190, part of the linker peptide (Witte et al., 1996).

The residues from both the light chain and the heavy chain were modelled in the MicroED structure (Fig. 2A). The linker between the light and heavy chain could not be resolved since the protease was in its active state.

Clostripain has a typical C11 protease structure as reported for other clostripain-like proteases (Gonzalez-Paez et al., 2019, McLuskey et al., 2016, Roncase et al., 2019, Roncase et al., 2017). Overall, clostripain consists of 15 alpha helices and 13 beta strands, with 2 alpha helices and 5 beta strands in the light chain and 13 helices and 8 beta strands in the heavy chain (Fig. 2B). The alpha helices and beta strands are numbered starting from the N-terminal region at residue 51 and ending in 526 at the C-terminal (Fig. 2B). The structure is made up of a central nine-stranded β-sheet typical of C11 proteases (Gonzalez-Paez et al., 2019, McLuskey et al., 2016, Roncase et al., 2019, Roncase et al., 2017) forming an α/β/α sandwich architecture (Fig. 2B). The β-strands involved are β1–β5, β8–β10 and β12. Out of the nine beta strands, seven β-strands are parallel and two strands are antiparallel (β3 and β10) (Fig. 2B). Among the nine β-strands, β1–β5 belong to the light chain, and the remaining four are in the heavy chain (Fig. 2). The alpha helices surrounding the beta sheet includes α1 (from light chain), α5 and α6 (from heavy chain) on one side and α2 (light chain) and α4 (heavy chain) on the other. There are two pairs of β-hairpins (β6 and β7, and β11 and β13) antiparallel to each other.

The active site of clostripain (Fig. 3A) consists of a catalytic dyad with the residues His176 and Cys231 (Labrou and Rigden, 2004). His176 belongs to the light chain and Cys231 belongs to the heavy chain, both of which are resolved in the density map (Fig. 3B).

Electrostatic surface analysis (Fig. 3C) shows that the catalytic dyad forms part of a large electronegative pocket consistent with a binding site for a positively charged substrate, confirming its arginine-specific cleavage and its potential to accommodate larger peptides. The pocket is also lined with the residue Asp229 (shown in Fig. 3A and 3C) which has been predicted to be the P1 specificity-determining residue in clostripain (Ullmann and Jakubke, 1994), similar to Asp177 shown in PmC11 previously (McLuskey et al., 2016).

### Comparison of the active clostripain structure with the AlphaFold model representing the inactive form

**Fig. 4:**
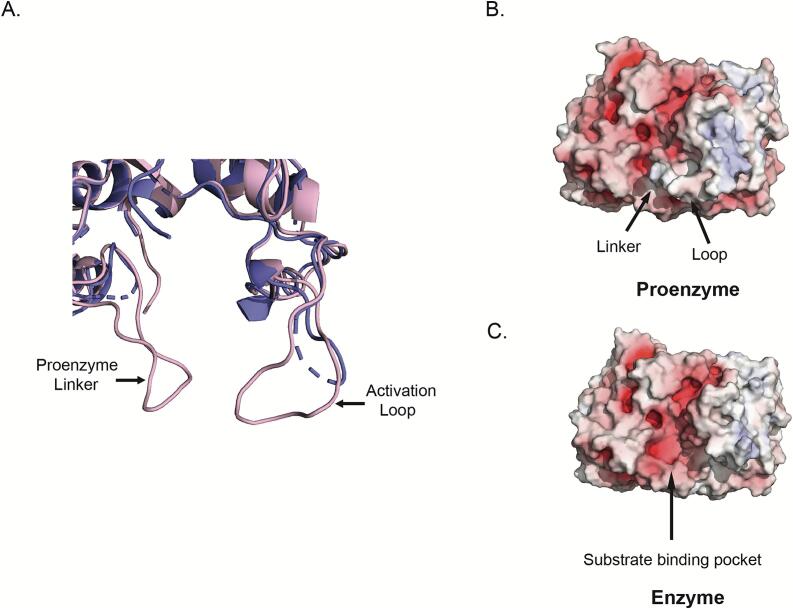
(A) Superposition of the MicroED structure (purple cartoon) and the AlphaFold model (light pink cartoon) of clostripain highlighting the linker and the loop. The linker and the loop formed by residues 452 – 457 are labelled on the proenzyme. (B) Electrostatic surface for the AlphaFold model representing the proenzyme. (C) Electrostatic surface for the active clostripain MicroED structure. Here blue and red denote positively and negatively charged surface potential, respectively, contoured at ±10 kT/e.

Superposition of the experimental MicroED structure with the predicted AlphaFold model highlights important functional differences. Superposition of the two structures (Fig. 4A) revealed that the structures differed with mainchain r.m.s.d of 0.57 Å. Strikingly, the AlphaFold model predicts the proenzyme structure while we determined the activated enzyme structure (Fig. 2). Comparison of the proenzyme model with the experimental structure of the active protease highlights the effect of the linker peptide on the substrate binding pocket. The electrostatic surface diagram of the proenzyme demonstrated that the linker between the light and heavy chain has an electropositive surface, and it gates the substrate binding pocket (Fig. 4B). Once the enzyme is activated, the linker is cleaved out therefore exposing the substrate binding pocket (Fig. 4C) to peptides. The removal of the gating peptide also changes the overall electrostatics making them more favorable for substrate binding.

Furthermore, we hypothesize that the loop formed by the residues 452 to 457 (STYYTS), (Fig. 4A) plays a significant role in substrate binding. In the proenzyme, the loop projects towards the active site and provides support to the linker to keep the active site closed (Fig. 4B). Absence of density for these residues in the MicroED structure could potentially mean that when the protease is activated and the linker removed, this loop becomes more dynamic and undergoes a structural rearrangement to accommodate substrate binding. A similar structural mechanism was previously described for metacaspase from *Trypanosoma brucei* (PDB ID: 4AF8), an arginine-specific C14 clan CD peptidase, which also requires a calcium ion for activation. In the presence of a calcium ion, it was shown that a conformational change occurs in loop 7 of the protease thus facilitating substrate binding (McLuskey et al., 2012). Consistent with the above reports this suggests that a similar mechanism may exist in clostripain.

### Structural comparison of clostripain with other C11 protease structures in the PDB

The structure of clostripain was compared with other C11 protease structures by a DALI structural similarity search (Holm and Rosenstrom, 2010). The top related structures include: clostripain-related protein from *B. thetaiotaomicron* (PDB ID: 6N9J, Z score = 33.3, r.m.s.d. = 2.4 Å, number of residues = 314/356, % ID = 20) (Roncase et al., 2019), inactive zymogen C11 protease from *Parabacteroides distasonis* (PDB ID: 6MZO, Z score = 32.2, r.m.s.d. = 2.7 Å, number of residues = 311/346, %ID = 21 (Gonzalez-Paez et al., 2019), cysteine proteases from *B. fragilis* (PDB ID: 5DYN, Z score = 18.5, r.m.s.d. = 2.6 Å, number of residues = 220/245, %ID = 19) (Choi et al., 2016) and *Parabacteroides merdae* (PDB ID: 3UWS, Z score = 18.2, r.m.s.d. = 2.6 Å, number of residues = 216/228, % ID = 20) (McLuskey et al., 2016), and gingipain from *Porphyromonas gingivalis* (PDB ID: 1CVR, Z score = 11.4, r.m.s.d. = 3.7 Å, number of residues = 179/433, %ID = 9) (Eichinger et al., 1999).

**Fig. 5:**
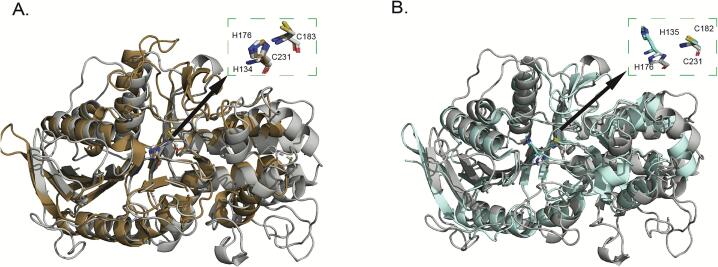
Superposition of clostripain (gray cartoon) with (A) Clostripain-related protein from *B. thetaiotaomicron* (PDB ID: 6N9J; brown cartoon), (B) inactive zymogen C11 protease from *Parabacteroides distasonis* (PDB ID: 6MZO; cyan blue cartoon). The active site residues are shown in the insets.

The structure of clostripain was superposed with the two structures of highest similarity (PDB ID: 6N9J and 6MZ0) as shown in Fig. 5. Overall, the structures share similar central α/β/α sandwich architecture. As seen in Fig. 5A, the active site residues His176 and Cys231 of clostripain superimpose with His134 and Cys183 and have highly conserved spatial orientation. The distance between Cys-His of clostripain overlapping with the inactive zymogen protease (Fig. 5B) shows similar structural features, except that the His residue, His135 in 6MZO is closer to the core (Gonzalez-Paez et al., 2019) and orients differently to His176 in clostripain. The distance between Cys-His of clostripain is 5.6 Å/5.2 Å, comparable to other cysteine proteases but less than 6 Å and 5.7 Å observed in the protease from *B. thetaiotaomicron* and the inactive zymogen protease.

## Conclusion

Here we report the previously unknown structure of a C11 protease, clostripain from *C. histolyticum* in its active form determined by MicroED. While the structure of clostripain was unattainable by other methods, MicroED delivered a 2.5 Å resolution structure using a single nanocrystal. This study adds to the growing list of novel macromolecular structures determined by MicroED and can form the basis for future development of new protease inhibitors. Future work on substrate binding in a time-resolved manner may provide additional insight into the mechanistic basis of substrate specificity and mode of activation of this protease.

## Materials and methods

### Materials

Clostripain (from *C. histolyticum*) in lyophilized, pre-activated form was purchased from Abnova (Taiwan) and used without further purification. Crystallization screens were purchased from Hampton Research (Aliso Viejo, CA). All reagents were made with MilliQ water. Tris buffer saline (TBS) was purchased from BioRad.

### Crystallization

Clostripain was solubilized in 1x TBS at a concentration of 10 mg/ml for setting up crystallization plates. Crystallization screens were set by sitting drop vapor diffusion method using a Mosquito crystallization robot, in 200 nl drops, at 1:1 sample to mother liquor ratio in 96-well Intelliplates (Hampton Research). Initial needle-shaped crystals were formed within 2 to 3 days at 20 °C in presence of 0.2 M ammonium phosphate monobasic, 0.1 M TRIS pH 8.5, 50 % v/v 2-methyl-2,4-pentanediol. Seeding stock was prepared using the protocol specified in the seed bead steel kit HR4-780 (Hampton Research). The seeding stock was used to make robust crystals in 24-well plates by hanging drop vapor diffusion in the mixture of 1 μl protein, 0.1 μl the seeding stock and 1 μl crystallization condition of 0.2 M ammonium acetate, 0.1 M Na-citrate tribasic dihydrate pH 5.6, 30 % w/v PEG 4000. The plates were kept at 20 °C and crystal growth was observed in 1 day.

### Sample preparation

The EM grids with clostripain crystals were prepared in a Leica GP2 plunge freezer set to 95 % humidity and 20 °C temperature as described previously (Martynowycz et al., 2019). Quantifoil Cu 200 R2/2 holey carbon grids (Quantifoil) were glow-discharged negatively for 45 s before sample application. The crystal drops were then diluted with 2 μl of crystallization condition and applied to the grids. The grids were each blotted for 20 s, then plunge-frozen into liquid ethane and stored in liquid nitrogen until use. The grids were clipped prior to screening in the electron microscope.

### FIB milling of the crystals

Clipped grids containing clostripain crystals were then loaded into a Aquilos dual-beam FIB/SEM (Thermo Fisher) operating at −180 °C following procedures described previously (Martynowycz et al., 2019). The grids were sputter-coated with a thin layer of platinum to preserve the sample during imaging and ion beam milling. Complete atlases of the grids were acquired using the MAPS software (Thermo Fisher). The milling sites were located and the eucentric height adjusted. The crystals were then milled using a gallium ion beam. The current used for milling was gradually decreased from 0.5 nA to 30 pA with every reduction in the thickness of the lamella. Milling was stopped when lamellae of desired thickness 300 nm were obtained.

### MicroED data collection

After milling, the grids with the milled lamella were transferred to a Titan Krios G3i TEM (Thermo Fisher) operating at −190 °C and an accelerating voltage of 300 kV (∼0.0197 Å wavelength). The Krios is equipped with a field emission gun and a Falcon 4i direct electron detector. The software EPU (Thermo Fisher) was used to acquire a low magnification atlas of the whole grid to identify the lamellae. The stage position was moved to each lamella and the eucentric height then adjusted by taking live view in Velox (Thermo Fisher). A Selectris energy filter operating at a slit width of 20 eV was used for the data collection. The selected area (SA) aperture (∼2 μm in diameter) was inserted and centered on the desired area to obstruct any background reflections. Initial screening of the lamellae for diffraction was carried out using Velox. Lamellae showing quality diffraction spots were used for data collection. MicroED data were collected by continuous rotation at a rate of 0.07°/s for 420 s using SerialEM. The sample-to-detector distance was set to the calibrated distance of 2941 mm. The data were collected using continuous rotation method with a Falcon 4i direct electron detector in counting mode (Martynowycz et al., 2022).

### MicroED data processing

The diffraction data were converted to SMV format using the MicroED tools (https://cryoem.ucla.edu/downloads/snapshots) (Hattne et al., 2015, Martynowycz et al., 2019). The diffraction datasets were indexed and integrated in XDS (Kabsch, 2010). Integrated intensities were scaled using XSCALE (Kabsch, 2010). Molecular replacement was carried out in Phaser (McCoy et al., 2007) using the AlphaFold model (A0A4U9RR22) available in the AlphaFold database as the template. Structure refinement and modelling were carried out in phenix.refine (Afonine et al., 2012) and Coot (Emsley and Cowtan, 2004), respectively. PyMOL (Schrodinger) was used to generate figures. Figures were assembled in Adobe Illustrator.

### CRediT authorship contribution statement

**Yasmeen N. Ruma:** Writing – review & editing, Software, Methodology, Formal analysis, Data curation, Writing – original draft, Conceptualization, Investigation. **Guanhong Bu:** Writing – review & editing, Methodology, Data curation. **Johan Hattne:** Writing – review & editing, Validation, Data curation, Software. **Tamir Gonen:** Writing – review & editing, Validation, Supervision, Resources, Project administration, Funding acquisition, Conceptualization.

## Declaration of competing interest

The authors declare the following financial interests/personal relationships which may be considered as potential competing interests: Tamir Gonen reports financial support was provided by National Institute of Health. Tamir Gonen reports financial support was provided by Howard Hughes Medical Institute. If there are other authors, they declare that they have no known competing financial interests or personal relationships that could have appeared to influence the work reported in this paper.

## Data Availability

Coordinates and maps were deposited in the Protein Data Bank (accession code 9CIP) and the Electron Microscopy Data Bank (accession code EMD-45623).
